# Case report: Symmetrical and increased lateral sway-based walking training for patients with corpus callosum infarction: a case series

**DOI:** 10.3389/fneur.2024.1330975

**Published:** 2024-06-24

**Authors:** Ying Jiang, Sijing Chen, Dan Wu, Wei He, Xiaoqing Ma, Lixia Zhang, Qian Zhang

**Affiliations:** ^1^Department of Rehabilitation, Jiangsu Zhongshan Geriatric Rehabilitation Hospital, Nanjing, Jiangsu, China; ^2^Department of Rehabilitation, The First Affiliated Hospital of Nanjing Medical University, Nanjing, Jiangsu, China; ^3^Department of Rehabilitation, The Second People's Hospital of Lishui, Lishui, Zhejiang, China; ^4^Department of Rehabilitation, Rehabilitation Hospital Affiliated to Nanjing Institute of Physical Education, Wuxi, Jiangsu, China; ^5^Department of Rehabilitation, The Geriatric Hospital Affiliated to Nanjing Medical University, Nanjing, Jiangsu, China

**Keywords:** stroke, corpus callosum, lower extremity, case report, walking

## Abstract

**Introduction:**

Corpus callosum injury is a rare type of injury that occurs after a stroke and can cause lower limb dysfunction and a decrease in activities of daily living ability. Furthermore, there are no studies that focus on the progress in rehabilitation of the lower limb dysfunction caused by infarction in the corpus callosum and the effective treatment plans for this condition. We aimed to present a report of two patients with lower limb dysfunction caused by corpus callosum infarction after a stroke and a walking training method.

**Methods:**

We implemented a walking training method that prioritizes bilateral symmetry and increases lateral swaying before the patients established sitting/standing balance. The plan is a rapid and effective method for improving walking dysfunction caused by corpus callosum infarction.

**Case characteristics:**

Following sudden corpus callosum infarction, both patients experienced a significant reduction in lower limb motor function scores and exhibited evident gait disorders. Scale evaluations confirmed that walking training based on symmetrical and increased lateral sway for patients with lower limb motor dysfunction after corpus callosum infarction led to significant symptom improvement.

**Conclusion:**

We report two cases of sudden motor dysfunction in patients with corpus callosum infarction. Symmetrical and increased lateral sway-based walking training resulted in substantial symptom improvement, as confirmed by scale assessments.

## 1 Introduction

Stroke is a common cardiovascular disease, with sequelae including limb movement disturbance, sensory disturbance, and cognitive disturbance, which impair a patient's ability to live independently, affecting their return to family and social life (1, 2). The corpus callosum, located at the bottom of the interhemispheric fissure on both sides of the cerebral hemisphere, is the largest white matter structure in the brain. Its main function is to maintain independent processing and integration of information between the two hemispheres (3, 4). Infarction in the corpus callosum region is relatively rare clinically, accounting for about 2.9–8% of ischemic strokes (5–7). This is due to the rich blood supply in the corpus callosum region, as blood supply is maintained from both the anterior and posterior circulations (8).

The clinical manifestations of corpus callosum infarction are complex and varied, including limb paralysis, ataxia, sensory disturbances, facial paralysis, and hemianopsia (9). Lower limb dysfunction can present as weakness in the lower limbs, unstable sitting posture, and difficulties in standing and walking (10). Patients with corpus callosum infarction sometimes find it more challenging to establish sitting and standing balance and regain community ambulation compared to regular hemiplegic patients. Incidentally, we observed that the effect of conventional methods on walking dysfunction caused by corpus callosum injury is not ideal, imposing a significant burden on patients, their families, and society. Therefore, there is a need to study rehabilitation training methods specifically for walking dysfunction caused by corpus callosum infarction.

However, current research on corpus callosum infarction mainly focuses on its complex clinical manifestations and imaging features (5, 9–14). Effective rehabilitation methods for walking dysfunction caused by corpus callosum infarction are still lacking. In this case report, we aimed to present a report of two patients with lower limb dysfunction caused by corpus callosum infarction after the occurrence of a stroke. These patients showed poor sitting balance and could not walk autonomously; however, after they were given walking training based on symmetrical and increased lateral sway, they achieved indoor short-distance walking in a short time, in addition to maintaining stable walking and attaining normal gait in 1 week. They went on to retain their good independent walking and living ability when they were discharged from the hospital.

## 2 Case descriptions

### 2.1 Patient 1

A 72-year-old woman was admitted to our hospital after experiencing weakness of the right limb and slurred speech for 25 days. She had a history of hypertension for 5 years and was prescribed amlodipine tablets. Upon physical examination, the patient demonstrated normal consciousness and cooperation. She completed three steps of the listening comprehensive test with no obvious abnormalities in comprehension. In the sitting or standing position, she could not maintain balance even with assistance (severe trunk tilting to the right). The Brunnstrom grade of the right limb was VI-VI-II (upper limb-hand-lower limb). The modified Ashworth scale of the right limb was measured as zero. Myodynamia of the right upper limb was above 4/5 and that of the lower right limb was below 2/5. There was no abnormality in superficial and deep somatic sensations. The right-side tendon reflexes were all reduced, and the Babinski sign was passive. The Holden grade (HG) was 0, indicating that she could not walk; modified Barthel ADL index (BI) was 10, indicating severe functional impairment; and Mini-Mental State Examination Score was 27, indicating normal cognition. The patient's medication history included aspirin enteric-coated tablets 100 mg, qd; clopidogrel bisulfate tablets 75 mg, qd; atorvastatin 20 mg, qd; potassium chloride sustained-release tablets 500 mg, bid; sertraline hydrochloride tablets 50 mg, qd; and amlodipine besylate tablets 5 mg, qd.

**Figure 1:**
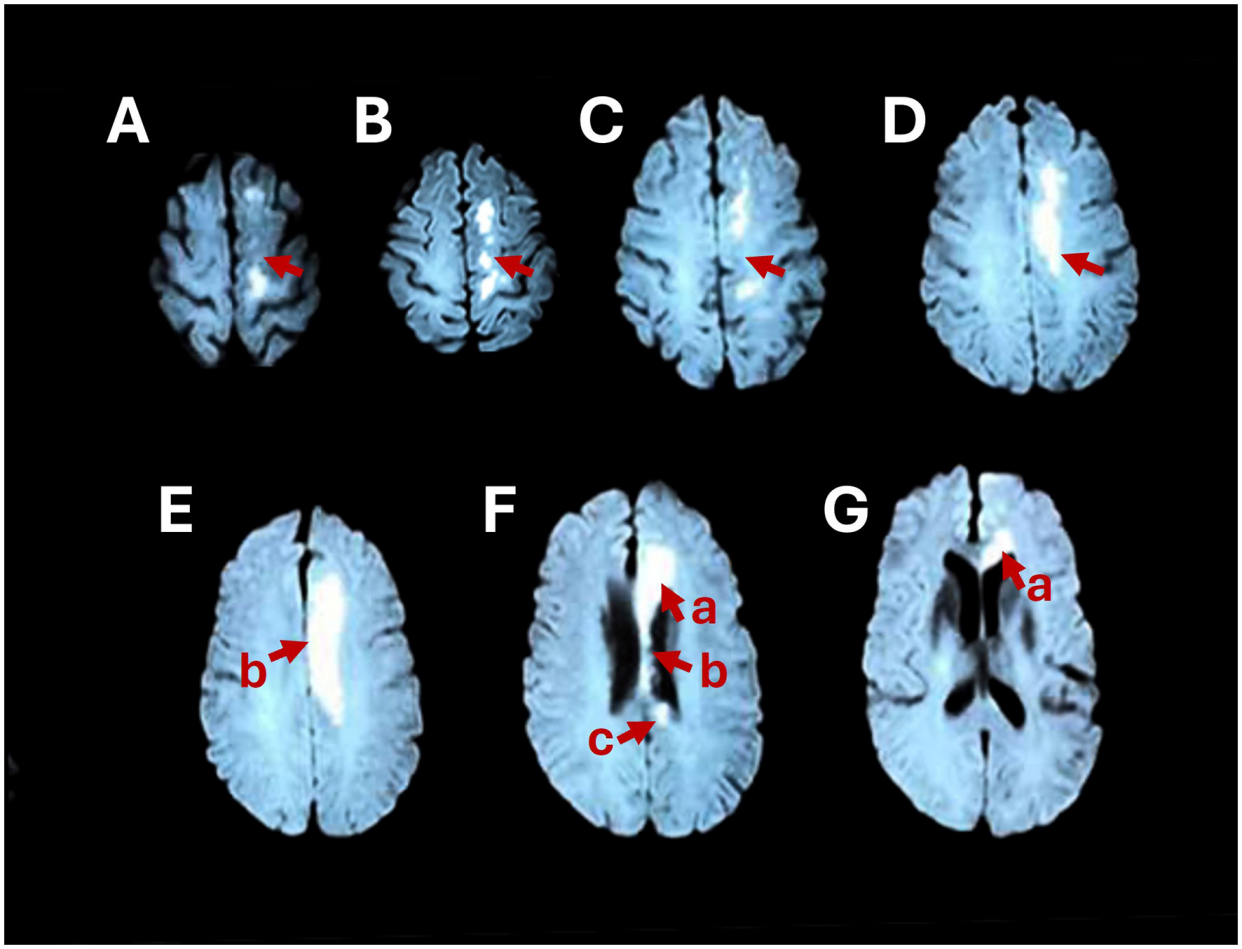
Brain MRI shows abnormally high signals in the left centrum semiovale **(A–D)** and the genu **(F**-a**, G**-a**)**, body **(E**-b**, F**-b**)**, and splenium **(F**-c**)** of the corpus callosum on axial DWI.

Magnetic resonance imaging (MRI) of the head revealed that the left centrum semiovale and the genu, body, and splenium of the corpus callosum were damaged (Figure 1). We diagnosed cerebral infarction convalescence. Furthermore, cerebral angiography plus internal carotid artery implantation revealed hypertension grade 3 (extremely high risk) and right carotid artery stenosis.

### 2.2 Patient 2

A 48-year-old woman was admitted to our hospital after left limb weakness, an inability to sit up, communication disorder, and unresponsiveness for 22 days. She had a history of hypertension for more than 20 years, for which she had been taking losartan hydrochlorothiazide tablets and nifedipine sustained-release tablets. Mental disorder and depression were diagnosed 9 years prior and related drugs were not taken regularly. She had type 2 glycosuria, diagnosed 1 month prior, and metformin hydrochloride sustained-release tablets and miglitol were taken orally.

Upon physical examination, the patient demonstrated normal consciousness, listening comprehension disorder, poor visual understanding, low voice, and diminished speech. In the sitting position, she could maintain balance and touch objects in different directions. In the standing position, she could not maintain balance, even with assistance. The Brunnstrom grade of the left limb was IV-III-II (upper limb-hand-lower limb); modified Ashworth scale measured as zero. Myodynamia of the left upper limb was above 3/5 and that of lower left limb below 2/5. Sensory examination could not be performed because of poor cooperation. The tendon reflexes left were normal, and the Babinski sign was passive. HG was 0, indicating that she could not walk, and BI was 0, indicating severe functional impairment. The patient's medication history included aspirin 100 mg, qd; nifedipine controlled release tablets 30 mg, qd; losartan hydrochlorothiazide tablets 1 pill, qd; donepezil hydrochloride 10 mg, qd; metformin hydrochloride sustained-release tablets 500 mg, bid; and miglitol 50 mg, tid.

**Figure 2:**
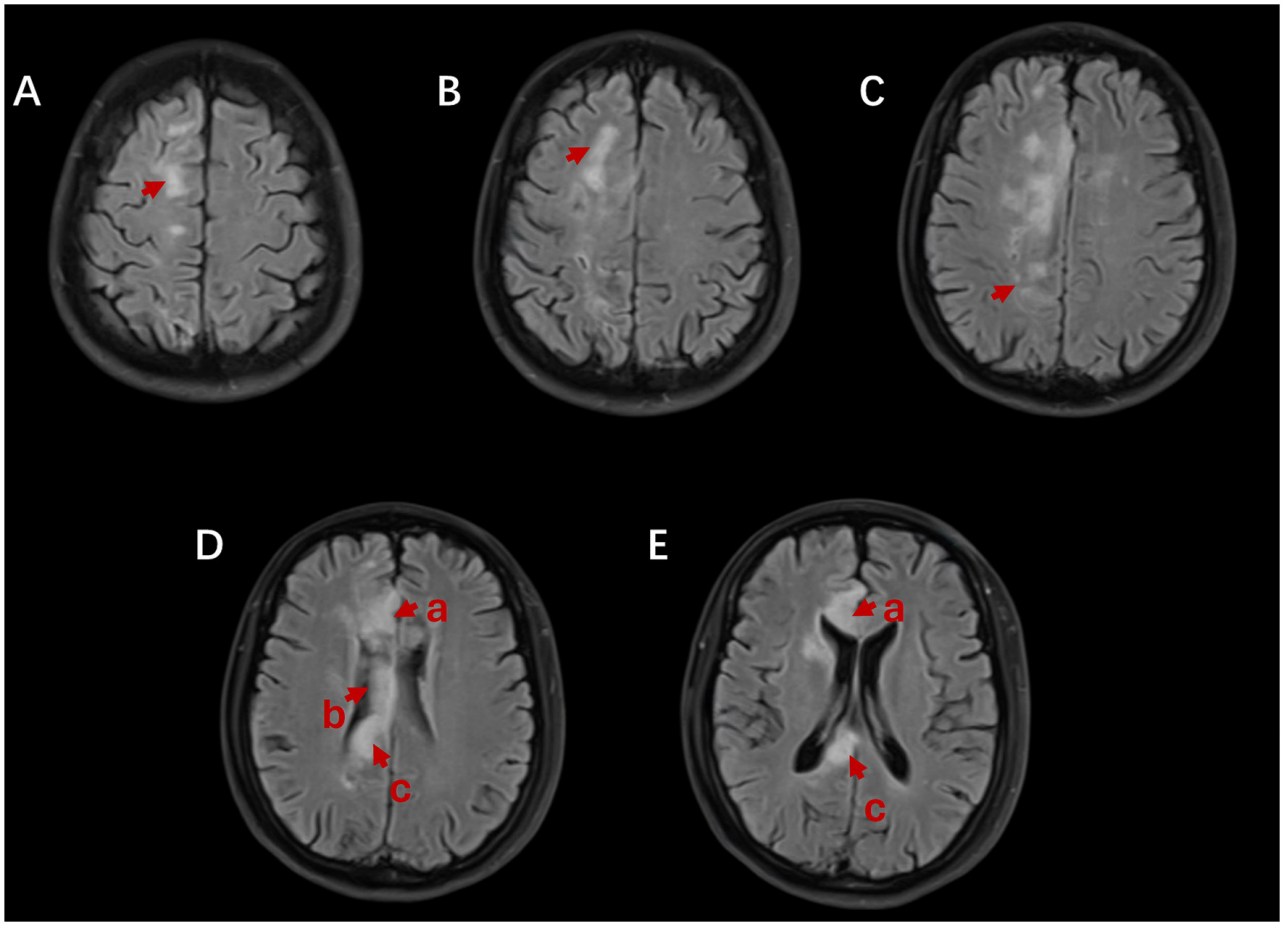
Brain MRI shows abnormally high signals in the right frontal lobe **(A, B)**, parietal lobe **(C)**, and the genu **(D**-a, **E**-a**)**, body **(D**-b**)**, and splenium **(D**-c**, E**-c**)** of the corpus callosum on axial fluid-attenuated inversion recovery (FLAIR).

The head MRI revealed the right frontal lobe, parietal lobe, and genu, body, and splenium cerebral infarction in the corpus callosum (Figure 2). We diagnosed cerebral infarction convalescence, left limb dysfunction with cognitive impairment, hypertension grade 3, and type 2 diabetes mellitus.

## 3 Diagnostic assessments

At the first assessment, Patient 1 could not stand by herself, and her body tilted severely to the right while walking. The therapist adopted walking training with symmetrical and increased lateral sway, holding the patient from behind with his arms passing underneath the armpits. When the patient showed dumping to the right (affected side), the therapist swayed the patient's trunk to the left at the moment when the patient was about to fall, increasing the amplitude of the sway symmetrically with the amplitude of the right sway. Then, the therapist would step to guide the patient to step on her right foot. After the right foot landed, the therapist followed the patient to step on her left foot. After the left foot landed, the patient dumped to the right again, and the steps for walking training would be repeated. The therapist was behind the patient during the whole walking process. This allowed the patient to better feel the shift of the center of gravity during walking, the coordination of walking, and the entire gait cycle. The training frequency was 40 min, twice per day, 6 times per week. The patient was also treated with physical therapy and occupational therapy.

After 3 days, Patient 1 could walk indoors for a short distance, and within 1 week, she could walk independently. After 2 months of treatment, the patient was discharged from the hospital. The Brunnstrom grade of the right limb was VI-VI-VI (upper limb-hand-lower limb); BI, 100; HG, 5/5,3 m timed-up-and-go test, 9.14 s; 10 m walk test, 17.52 s; 6 min walk test, 350 m; sitting balance grade, 3/3; and standing balance grade, 3/3. A follow-up telephone survey after 3 months showed that the patient's community walking ability was grade 5, indicating that she could walk independently in the community environment and do simple housework such as sweeping the floor, mopping the floor, washing dishes, and folding clothes.

We adopted the same method for Patient 2. Because this patient with corpus callosum infarction showed dumping to the left (affected side), the therapist swayed the patient's trunk to the right at the moment when the patient was about to fall, increasing the amplitude of the sway symmetrically with the amplitude of the left sway. Then, the therapist stepped to guide the patient to step on her left foot. After the left foot landed, the therapist followed the patient to step on her right foot. After the right foot landed, the patient dumped to the left again, and the steps were repeated. The training frequency was 30 min, twice per day, five times per week.

Patient 2 could walk with the help of two people after 2 days of treatment, with the help of one person in < 1 week, and completely independently after 2 weeks. After 28 days, the patient was discharged from the hospital. The Brunnstrom grade of the left limb was V-V-V (upper limb-hand-lower limb); BI, 70; HG, 4/5; sitting balance grade, 3/3; and standing balance grade, 3/3.

## 4 Discussion

Following sudden corpus callosum infarction, both patients experienced a significant reduction in lower limb motor function scores and exhibited evident gait disorders. Scale evaluations confirmed that direct assisted walking training for patients with lower limb motor dysfunction after corpus callosum infarction led to a significant improvement of symptoms.

The corpus callosum is the most critical fibrous bundle plate connecting the bilateral hemispheres and plays a crucial role in information acquisition, functional coordination, and reorganization between the bilateral hemispheres (15). The genu (“knee”) of the corpus callosum connects the prefrontal cortex with the higher sensory region, the middle of the body connects the primary and secondary somatosensory and motor regions, the posterior middle body is involved in transmitting information from the primary and secondary auditory regions, the pressure part connects the occipital visual region, and the isthmus (the junction of the body and the pressure part) connects the fibers of the motor, somatosensory, and primary auditory regions (16).

A retrospective study of 127 patients with corpus callosum infarction showed that 73.2% had limb paralysis, 38.6% had facial paralysis, 20.5% had sensory disturbance, 9.4% had hemianopia, and 7.9% had ataxia (9). Limb paralysis is manifested as monoplegia and hemiplegia (7) and weakness of both lower limbs (17). The mechanism of dysfunction caused by corpus callosum injury needs to be elucidated (5). Diseases that cause extensive functional changes in the connectivity between the prefrontal lobe and the hemisphere may lead to motor and attention deficits (18, 19); other studies have suggested that damage to the corpus callosum may lead to widespread disruption of motor and cognitive cortical activities, leading to a combination of attention and motor disorders in stroke patients (20).

There are two models of interhemispheric interaction after stroke that describe the possible neural mechanisms of recovery after brain injury: the compensatory and interhemispheric competition models (21). The former assumes that the residual motion network replaces the activity of the affected area, and the unaffected hemisphere will compensate for the function of the damaged area (22, 23). The competition model between hemispheres assumes that, after cerebral infarction, the inhibition of balance between hemispheres is destroyed; the inhibition of the damaged hemisphere to the healthy hemisphere is insufficient, while the inhibition of the healthy hemisphere to the damaged hemisphere is increased, resulting in the influence of the balance between bilateral hemispheres (24, 25). Thus, the function of the damaged hemisphere is excessively inhibited (26–28). This mechanism is mediated by the corpus callosum (29–31). Many studies have proven that the corpus callosum participates in the information interaction, integration, and motor coordination between hemispheres (32–35). However, corpus callosum lesions lead to asymmetric information of bilateral use and the treatment strategy for patients with corpus callosum injuries should involve the coordinated use of both sides.

We observed that the effect of treating walking dysfunction caused by corpus callosum injury by conventional methods is not ideal. Traditionally, stroke patients recover their walking and independent living ability through turning over, sitting up, sitting balance, sitting–standing conversion training, standing balance, and walking training. Relatively speaking, these activities, especially the early training for brain injury (turning over in bed, sitting up, sitting balance, sitting and standing balance), cannot fully mobilize the use of bilateral symmetry information involving the whole body. Therefore, it often takes a lot of time to treat lower limb dysfunction caused by corpus callosum injury according to conventional methods. However, patients may not quickly re-establish standing balance or acquire effective walking ability. Standard neurological rehabilitation and gait training are not always effective in restoring normal gait (36). An innovative approach is needed to treat dysfunction caused by corpus callosum infarction.

Because the corpus callosum is mainly responsible for the interaction and integration of information between bilateral hemispheres, which involves the coordination and cooperation of bilateral motor functions, a lesion in the corpus callosum may lead to the joint impairment of attention and movement disorders in stroke patients. Based on the characteristics of dysfunction caused by corpus callosum lesions, the rehabilitation treatment strategy needs to meet the following conditions: (1) The treatment must be completed under dynamic conditions to achieve reconstruction coordination; (2) Attention should be paid to the symmetry of the use of information in the treatment to correct the asymmetry of bilateral use information caused by the lesion of the corpus callosum; (3) During the treatment, it is necessary to mobilize the patient's attention mechanism and increase the patient's alertness. Therefore, we propose using dynamic daily life-related movements that can be symmetrical and mobilize bilateral limbs to cooperate with each other as a training movement, which may contribute to functional recovery after injury. The training of walking, a daily movement, meets the above requirements: walking requires bilateral limbs to cooperate and move with rhythm, symmetry, and coordination. This may promote the recovery of corpus callosum function more than static standing, sitting and standing, turning over, and sitting up. Therefore, we skip these basic movements and directly administer dynamic walking training. In addition, we speculate that walking training based on symmetrical and increased lateral sway is a challenging and unfamiliar new training task, which can enhance vigilance of attention and promote brain restructuring and walking for recovery (37). For patients with corpus callosum infarction, there are several main points in walking training based on symmetrical and increased lateral sway. First, this emphasizes the alternate symmetry of bilateral movements, which is based on the functional characteristics of the corpus callosum, and the symmetry treatment is more conducive to the symmetry activation of bilateral sensorimotor areas (38). Second, pay attention to the details of coordinated activities during walking: the therapist follows the patient and guides them to sway left and right, and back and forth, in sync with the walking cycle. The entire training emphasizes coordinated and rhythmic movement to assist the patient in completing walking. Additionally, the patient's body will naturally tilt toward the affected side. Rather than immediately correcting this tilt, the therapist should allow the patient to tilt to their maximum safe angle and then gently sway their trunk toward the healthy side. This keeps the patient in an unsteady environment, maintains proper alertness, improves attention, and strengthens the treatment input. In this state, the human body often mobilizes the unconscious proprioceptive pathway, which triggers the activation of the contralateral extensor muscle through a self-protection mechanism to keep standing (39, 40). The dorsiflexor and plantar flexor muscles of the affected side are activated to obtain greater sway and forward advancement ability (41). This new training method, which skips the routine training procedure, seems to be more effective for patients with corpus callosum infarction and can greatly shorten the walking training time and better restore the walking ability.

These two cases lack imaging evidence of the patients' brains, which makes it impossible to observe any changes in the brain. In the future, diffusion tensor imaging (DTI) technology can be employed to observe changes before and after treatment. This can provide insights into the recovery of damage in the corpus callosum area and explore potential mechanisms. Additionally, it is essential to note that corpus callosum infarction is a rare condition, and this case report includes only two patients. Therefore, our future direction of research is aimed at conducting more comparative studies between assisted walking and conventional treatment.

## 5 Patient perspective

Patient 1 expressed that during the initial stages of rehabilitation training, she was filled with concerns about the possibility of falling. However, with the careful assistance of the therapist, she gradually learned how to walk. Incredibly, in less than a week, she could walk independently and slowly. This left her feeling incredibly surprised and satisfied. Upon returning home, she could almost fully participate in family life, significantly boosting her sense of happiness.

Patient 2 initially struggled to communicate effectively with others, including family members. However, during the process of treatment, she actively cooperated and made significant progress. This left her family feeling very surprised and relieved. The efforts of Patient 2 and the effectiveness of the treatment allowed her to reintegrate into social life, bringing about significant changes for both her personally and her family.

## Data availability statement

The original contributions presented in the study are included in the article/supplementary material, further inquiries can be directed to the corresponding authors.

## Ethics statement

The studies involving humans were approved by the Ethics Committee of The Geriatric Hospital Affiliated to Nanjing Medical University. The studies were conducted in accordance with the local legislation and institutional requirements. The participants provided their written informed consent to participate in this study. Written informed consent was obtained from the individual(s) for the publication of any potentially identifiable images or data included in this article.

## Author contributions

YJ: Writing – review & editing, Writing – original draft. SC: Writing – review & editing, Writing – original draft. DW: Data curation, Writing – review & editing. WH: Writing – review & editing, Data curation. XM: Data curation, Writing – review & editing, Formal analysis. LZ: Writing – review & editing, Conceptualization. QZ: Writing – review & editing, Conceptualization.
